# Kaposiform hemangioendothelioma in tonsil of a child associated with cervical lymphangioma: a rare case report

**DOI:** 10.1186/1477-7819-9-57

**Published:** 2011-05-23

**Authors:** Bharat Rekhi, Shweta Sethi, Suyash S Kulkarni, Nirmala A Jambhekar

**Affiliations:** 1 Department of Pathology, Tata Memorial Hospital, Parel, Mumbai; 2 Department of Radiodiagnosis, Tata Memorial Hospital, Parel, Mumbai

## Abstract

Kaposiform hemangioendothelioma (KHE) is an uncommon vascular tumor of intermediate malignant potential, usually occurs in the extremities and retroperitoneum of infants and is characterized by its association with lymphangiomatosis and Kasabach-Merritt phenomenenon (KMP) in certain cases. It has rarely been observed in the head and neck region and at times, can present without KMP. Herein, we present an extremely uncommon case of KHE occurring in tonsil of a child, associated with a neck swelling, but unassociated with KMP. A 2-year-old male child referred to us with history of sore throat, dyspnoea and right-sided neck swelling off and on, since birth, was clinicoradiologically diagnosed with recurrent tonsillitis, including right sided peritonsillar abscess, for which he underwent right-sided tonsillectomy, elsewhere. Histopathological sections from the excised tonsillar mass were reviewed and showed a tumor composed of irregular, infiltrating lobules of spindle cells arranged in kaposiform architecture with slit-like, crescentic vessels. The cells displayed focal lumen formation containing red blood cells (RBCs), along with platelet thrombi and eosinophilic hyaline bodies. In addition, there were discrete foci of several dilated lymphatic vessels containing lymph and lymphocytes. On immunohistochemistry (IHC), spindle cells were diffusely positive for CD34, focally for CD31 and smooth muscle actin (SMA), the latter marker was mostly expressed around the blood vessels. Immunostaining for HHV8 was negative and Ki-67 (proliferation marker) displayed focal positivity. Diagnosis of KHE was made. Platelet count was towards lower side of range. Postoperative imaging showed discrete, multiple fluid containing lesions in the right neck that were high on T2-weighed sequences, on magnetic resonance imaging (MRI) and ipsilateral intraoral mucosal growth. Fine needle aspiration cytology (FNAC) smears from neck swelling showed blood, fluid and lymphocytes. Possibility of a coexisting lymphangioma was considered. The patient was offered sclerotherapy and is on follow-up. This case forms the second documented case of KHE at this site, along with its unique association with neck lymphangioma. KHE has distinct histopathological features and can be sorted out from its other differentials like juvenile hemangioma and Kaposi's sarcoma. IHC stains are useful in substantiating a definite diagnosis.

## Background

Kaposiform hemangioendothelioma (KHE), initially described by Zukerberg et al [1], is an intermediate/borderline vascular neoplasm between a hemangioma and a malignant angiosarcoma. It is a locally aggressive, rarely metastatic neoplasm, does not have a tendency for spontaneous regression and has characteristic histopathological features, including tumor cell architectural pattern resembling a Kaposi's sarcoma, along with lymphatic component, namely lymphangioma/lymphangiomatosis. In addition, it is known for its association with Kasabach-Merrittt phenomenon (KMP), a condition characterized by profound thrombocytopenia and life-threatening hemorrhage. These features differentiate this entity from a juvenile hemangioma that forms the closest differential diagnosis. It is usually identified in infancy and first decade of life at sites like extremities and retroperitoneum and uncommonly in the head and neck region [1-4]. At times, KHE can occur without KMP [5]. It has rarely been documented in the tonsil, and to our knowledge, only 1 such case has been documented in the western literature [6].

Herein, we present an extremely uncommon case of Kaposiform hemangioendothelioma associated with neck lymphangiomas, but unassociated with KMP, in a 2-year-old male child, who presented with right-sided tonsillar enlargement and was clinicoradiologically diagnosed with tonsillitis. Postoperative imaging unraveled ipsilateral coexisting lymphangioma. The differential diagnoses of this unique case are discussed herewith.

## Case Presentation

A 2-year-old male child referred to us with history of swelling right side neck, associated with episodes of pain and swelling in his throat, since birth. One of the episodes was severe that led to acute dyspnoea and dysphagia that was clinicoradiologically diagnosed as a peritonsillar abscess, for which the patient underwent a right-sided tonsillectomy, elsewhere. There was no history of bleeding or hemoptysis. The excised biopsy specimen was submitted to us in form of paraffin blocks and slides, for review.

**Figure 1:**
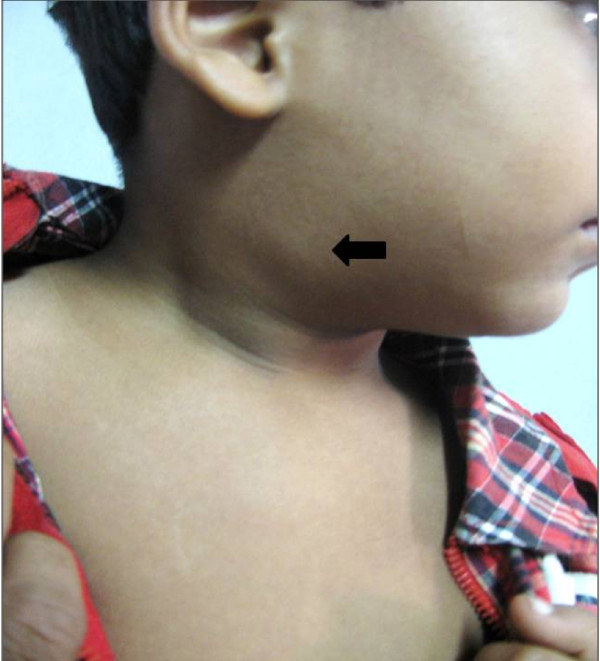
**Current clinical photograph of a swelling in the right side of neck (arrow), post tonsillectomy**.

Presently, his general condition was good. Clinically, a soft, mobile, cystic, right-sided neck swelling measuring 3 × 2 cm was noted. Figure 1. On oral examination, a 2 × 2 cm sized mucosal growth was noted with soft tissue enlargement in the right tonsillar area.

## Radiological Findings

**Figure 2:**
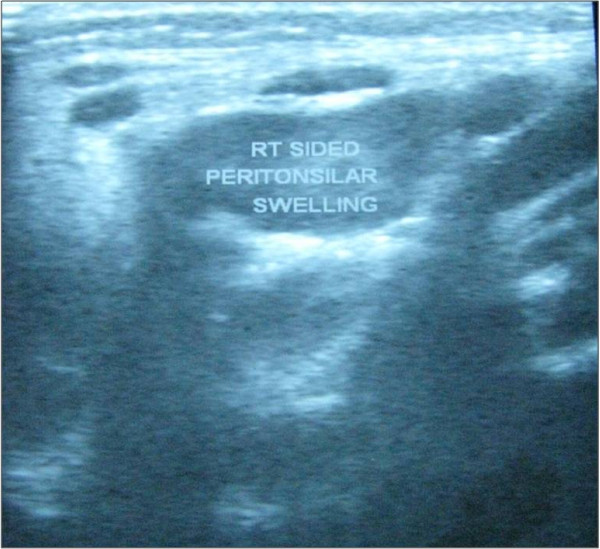
**Preoperative ultrasonography (USG) neck showing a tonsillar swelling in the right side**.

Preoperative ultrasonography (USG) neck revealed a swelling in the submandibular region and in posterior triangle of neck. These swellings were presumed to be lymph nodes. Diagnosis of an inflammatory lesion was considered. Figure 2.

**Figure 3:**
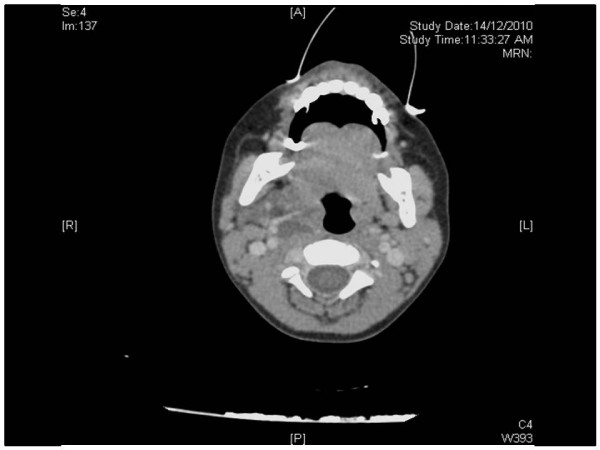
**Post operative computed tomography (CT) scan imaging showing multiple fluid containing rim enhancing lesions in the right side of neck**.

Postoperative plain and contrast computed tomography (CT) scan of head and neck region showed discrete, multiple fluid containing, rim enhancing lesions in right neck. These involved submandibular space and effaced right parapharyngeal fat planes. These distended cervical fascia, but did not breach to involve anterior cervical spine. Posteriorly, these were seen abutting carotid vessels inferiorly and extended nearly up to right thyroid. Ethmoid and maxillary sinuses were normal. There was no definite mass in the epiglottis that was otherwise bulky. Figure 3.

Post operative MRI (Magnetic resonance imaging) scan of neck and paranasal sinuses, using T1 and T2-weighed sequences in multiple planes revealed an ill defined predominantly hyperintense lesion on STIR and T2 weighed images in the right parapharyngeal space, containing fluid/blood, extending from C2 to C5 levels. It appeared hypointense to isointense on T1-weighed images and on intravenous administration of Gadolinium diethylenetriaminepentaacetic acid (Gd-DTPA), it showed peripheral enhancement. It measured approximately 4.3 × 2.3 × 3.6 cm. Anteriorly, the lesion extended up to submandibular region, posteriorly was in contact with longus capitis, laterally extended into the subcutaneous tissues of parotid gland, medially extended into the visceral neck space, superiorly reached up to inferior part of parotid and inferiorly, the lesion reached up to the right lobe of thyroid gland. Bilateral neck nodes (level II, III and V) were identified. Diagnosis of coexisting lymphangiomas was considered.

### Laboratory investigations

Haemoglobin was normal, 12.1 g/dl (Normal = 11-14 g/dl). Total leukocyte count (TLC) was normal. Differential leukocyte count (DLC) showed increase in eosinophils, 13.6% (Normal = 2-7%), as well as absolute count, 1.6456 × 10 e9/L (Normal = 0.2-1 × 10 e9/L). Platelet count was towards lower side of the range, 12.7 × 10^4^/μL (Normal = 13 to 37 × 10^4^/μL). Prothrombin time (PT) was high, 14. 9 sec (Normal = 10.8 -14.6 sec). Activated partial thromboplastin time (APTT) was towards higher side, 37.8 sec (Normal = 23-35 sec). International normalized ratio (INR) was normal, 1.2 (Normal = 0.8-.2). Serum uric acid levels were elevated 7.5 mg/dl (Normal = 3.5-7.2 mg/dl). Blood sugar was low, 55 mg/dl (Normal = 76-106 mg/dl).

## Pathological findings

As per referral gross description, an ovoid tissue measuring 1.7 cm diameter was processed for histopathological examination. It was reported as myofibromatosis, elsewhere and submitted to us for review.

### Histopathological findings

**Figure 4:**
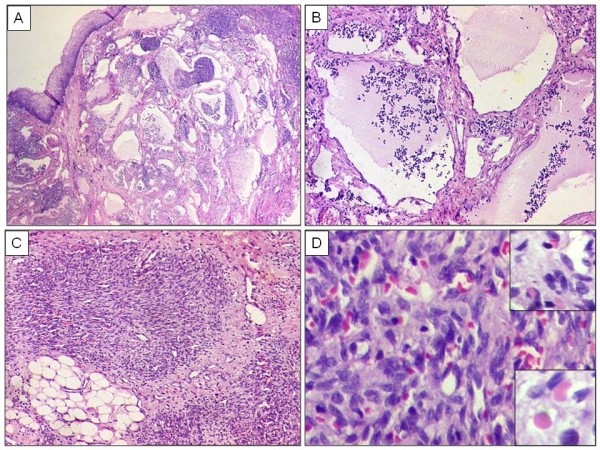
**Kaposiform hemangioendothelioma of tonsil**. A. Tonsillar epithelium with several dilated lymphatic spaces underneath reminiscent of lymphangioma along with nodules of spindle cells separated by fibrocollagenous stroma. H & E × 40. B. Higher magnification showing dilated lymphatic vessels containing lymph and lymphocytes. H & E × 200 C. Spindle cells in irregular fascicles with Kaposiform vascular pattern, slit-like vessels and extravasated red blood cells (RBC's). H & E × 200. D. Higher magnification showing slit-like crescentic capillaries within spindle cells, including single cells forming lumina and containing RBC's. H & E × 400. **Upper Inset **showing micro thrombi and eosinophilic bodies amid spindle shaped vascular cells. H & E × 1000. **Lower Inset **showing an eosinophilic body amid spindle cells. H & E × 1000.

Hematoxylin and Eosin (H & E) stained sections showed tonsillar epithelium with submucosal multiple, ill-defined, infiltrating nodules of spindle cells forming characteristic vascular pattern, separated by desmoplastic stroma. The tumor nodules were composed of criss-crossing spindle cell fascicles with interspersed capillaries that showed slit-like, crescentic lumens. In addition, there were extravasated red blood cells (RBC's), single cells with lumina containing RBC's, fibrin thrombi and eosinophilic globules. There was mild nuclear variation, but no significant nuclear atypia, mitosis or necrosis. Besides, there were discrete foci of several dilated lymphatic vessels containing lymph and lymphocytes within the submucosa. Figure 4 (A, B, C, D).

**Figure 5:**
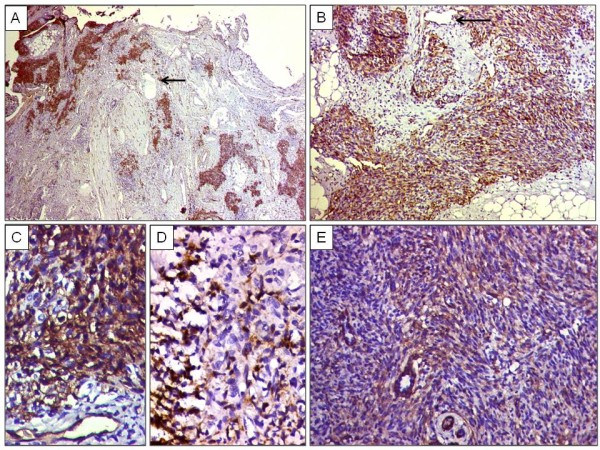
**Immunohistochemical results**. A. CD34 positivity within infiltrating tumor nodules separated by desmoplastic stroma and negativity in lymphatic vessels (arrows). 3'-3'-diaminobenzidine tetrahydrochloride. (DAB) × 40. B. Nodules of infiltrating spindle cells showing immunoreactivity to CD34. A vessel showing CD34 positivity is noted (arrow). DAB × 200. C. Higher magnification showing diffuse positivity with CD34. DAB × 400.D. CD31 positivity discretely within spindle-shaped tumor cells. DAB × 400.E. Focal SMA positivity within pericytic cells. DAB × 200.

On immunohistochemistry (IHC), the spindle cells were diffusely positive for CD34. CD31 was discretely positive in spindle cells. Smooth muscle actin (SMA) was focally positive, while Human Herpes virus (HHV)-8 staining was negative. MIB1 highlighted occasional tumor cells. The areas comprising several dilated lymphatic vessels showed negative staining with CD34 and CD31. Figure 5 (A, B, C, D, E). Diagnosis of Kaposiform hamenagioendothelioma was made. In view of lack of submission of other sections, status of resection margins could not be commented upon and presumably, it was an incomplete resection.

Postoperative fine needle aspiration cytology (FNAC) smears from the ipsilateral cervical lesion showed presence of blood, fluid and lymphocytes. In view of imaging findings, diagnosis of a coexisting ipsilateral neck lymphangioma was made.

The patient was offered sclerotherapy and is on follow-up.

## Discussion

The present case is the second documented case of Kaposiform hemangioendothelioma (KHE) in the right tonsil of a 2-year-old child, who referred to us with a clinical history of episodes of tonsillitis with ipsilateral neck swelling, since birth.

During one of the clinical episodes, the patient had acute dysphagia and dysponea, wherein he was radiologically diagnosed with a peritonsillar abscess and therefore, underwent right-sided tonsillectomy, elsewhere. On review of histopathology slides, the differential diagnoses included a juvenile hemangioma, Kaposi's sarcoma, myofibromatosis and hemangiopericytoma. Presence of irregular, infiltrating lobules of spindle cells with a "kaposiform" pattern, forming slit-like, crescentic capillaries with platelet thrombi, eosinophilic bodies and prominent areas of lymphangiomatosis were helpful in differentiating it from a juvenile hemangioma [1,2]. However, fortunately, the present case was not associated with KMP. Although, the platelet count was towards lower side of range, the patient did not present with features of life threatening thrombocytopenia and or anaemia. Even though a Kaposiform hemangioendothelioma is known to occur with KMP, this association has been noted in 42% cases, in a study by Lyons et al [2], wherein the authors documented 8 cases in the head and neck region, including 4 cases associated with KMP, while 3 cases unassociated with KMP. None of the cases in that study was noted in the tonsil region. KMP is more commonly seen in cases occurring in abdominal than somatic sites. Lately, Gruman et al [5] have also documented 10 cases of Kaposiform hemangioendothelioma, unassociated with KMP, including 3 cases in head and neck region, but none in the tonsil. Despite a Kaposiform pattern of tumor cells, including eosinophilic bodies, a Kaposi's sarcoma was ruled out in view of presence of several dilated lymphatic channels, focal capillary formation, lack of nuclear atypia and mitosis within tumor cells, along with HHV8 negativity. This reinforces lack of a common pathway for a Kaposiform hemangioendothelioma and a Kaposi's sarcoma. In spite of SMA positivity, aforementioned histological features and diffuse CD34 immunoreactivity and focal CD31 positivity within tumor cells, ruled out a myofibromatosis that has been documented at this site and in similar aged patients [7]. Infact, the present case was initially reported as myofibromatosis at another laboratory. Variable SMA positivity within tumor cells, presumably in the pericytes, has been documented in a KHE [2]. This reinforces application of an optimal panel of IHC markers with the already described histomorphological 'clues' for a KHE. Additional IHC markers like isoform 1, GLUT-1, a glucose transporter protein and Lewis Y antigen (LeY) have been found useful in differentiating KHE from a juvenile hemangioma (JH), as these are not expressed in KHE, in contrast to a juvenile hemangioma [2,8]. Ki-67 was noted in few tumor nuclei as similarly described by Lyon et al [2], who noted a contrasting prominent staining in cases of JH.

Presence of several co-existing dilated lymphatic vessels was a significant 'clue' in diagnosis of a KHE. It has been documented that approximately two-thirds of KHE, when carefully studied, exhibit lymphatic abnormalities comprising thin-walled vessels that surround vascular tumor nodules and often extend outward. One of the reasons that have been hypothesized for this association is that the development of KHE begins with a lymphatic malformation onto which a vascular component is engrafted. Another hypothesis is that KHE initially produces lymphatic endothelial growth factors (for example VEGF-C), that leads to proliferation of adjacent lymphatics, as noted in other tumors [2,9]. Site-wise, tonsil, as noted in the present case, seems to be a "fertile soil" for the development of this unusual tumor, with vascular and lymphatic components. Lately, D-240 has been identified as a useful marker for highlighting lymphatic endothelial cells [10]. However, in view of present unavailability of this marker in our laboratory, it was not included in the IHC panel. Nonetheless, histopathological features were unequivocal for presence of substantial lymphatic component, wherein the lymphatic channels were negative for CD34 and CD31, in contrast to the lobules of spindle cells [2]. Aforementioned histological features and lack of KMP in the present case were overlapping with a tufted hemangioma [11]. A similar co-existence of lymphangiectasia with vascular tumor nodules is seen in a tufted angioma. KHE and tufted angioma are probably same part of the spectrum. Cases of an acquired tufted angioma have been described with KMP, as well as cases of KHE have been described without KMP [5,12]. The platelet count in the present case was towards lower side of the range, but no symptoms of coagulopathy were noted, excluding a KMP.

Interestingly, on postoperative imaging in the present case, coexisting lymphangioma was also identified. This was a discrete lesion in the parapharyngeal region, excluding the possibility of the extension from the main lesion. This could possibly have been additional reason for transient ipsilateral neck swelling, since birth, reflective of episodic secondary inflammation.

Therapeutically, KHE, in isolation, is a candidate for complete surgical excision. Increasing size, risk of coagulopathy are indicators for therapeutic interventions in such cases. Medical treatment is included in cases associated with KMP [13]. KMP was lacking in the present case. Cases of KHE, unassociated with KMP have been followed-up without treatment and have shown no disease and even tumor regression in a few such cases [5]. Surgical excision in this case was performed elsewhere, presumably without clear resection margins, as a result of preoperative clinicoradiological impression of an inflammatory lesion. In view of postoperative imaging results that showed cystic lesion, indicative of coexisting lymphangiomas, the patient was offered sclerotherapy at our hospital. He has been recommended for 4 cycles of sclerotherapy on a 2 monthly basis.

**In conclusion**, KHE is an uncommon tumor with a distinct clinicopathologic features, including IHC profile and differs from a Kaposi's sarcoma and its other histological mimics. Careful attention towards its histopathological features, including its association with lymphatic component, coupled with IHC, is helpful in its identification, including at rare sites like tonsil in the present case. A coexisting lymphangiomas was a unique feature that led to incorporation of sclerotherapy in the present case. Surgical excision with follow-up is the treatment mainstay in most cases.

## Consent

Written informed consent was obtained from the patient for publication of this case report and any accompanying images.

## Competing interests

The authors declare that they have no competing interests.

## Authors' contributions

BR: Diagnosing pathologist, procured clinical details, collected references, prepared manuscript, artwork, did final editing of the manuscript. SS: Senior resident involved in diagnosis, collected some references. SK: Provided additional treatment details and post operative imaging results. NAJ: Diagnosis, overall supervision and gave approval. All authors have read and approved the final manuscript
